# Risk factors for exacerbations and pneumonia in patients with chronic obstructive pulmonary disease: a pooled analysis

**DOI:** 10.1186/s12931-019-1262-0

**Published:** 2020-01-06

**Authors:** Benjamin F. Hartley, Neil C. Barnes, Sally Lettis, Chris H. Compton, Alberto Papi, Paul Jones

**Affiliations:** 1 Veramed Ltd, Twickenham, UK; 2 GlaxoSmithKline plc, Brentford, UK; 3 William Harvey Institute, Bart’s and the London School of Medicine and Dentistry, London, UK; 4 GlaxoSmithKline plc, Uxbridge, UK; 5 University of Ferrara, Ferrara, Italy; 6 Institute of Infection and Immunity, St George’s, University of London, London, UK

**Keywords:** Chronic obstructive pulmonary disease, Exacerbation, Pneumonia, Meta-analysis

## Abstract

**Background:**

Patients with chronic obstructive pulmonary disease (COPD) are at risk of exacerbations and pneumonia; how the risk factors interact is unclear.

**Methods:**

This post-hoc, pooled analysis included studies of COPD patients treated with inhaled corticosteroid (ICS)/long-acting β_2_ agonist (LABA) combinations and comparator arms of ICS, LABA, and/or placebo. Backward elimination via Cox’s proportional hazards regression modelling evaluated which combination of risk factors best predicts time to first (a) pneumonia, and (b) moderate/severe COPD exacerbation.

**Results:**

Five studies contributed: NCT01009463, NCT01017952, NCT00144911, NCT00115492, and NCT00268216. Low body mass index (BMI), exacerbation history, worsening lung function (Global Initiative for Chronic Obstructive Lung Disease [GOLD] stage), and ICS treatment were identified as factors increasing pneumonia risk. BMI was the only pneumonia risk factor influenced by ICS treatment, with ICS further increasing risk for those with BMI <25 kg/m^2^. The modelled probability of pneumonia varied between 3 and 12% during the first year. Higher exacerbation risk was associated with a history of exacerbations, poorer lung function (GOLD stage), female sex and absence of ICS treatment. The influence of the other exacerbation risk factors was not modified by ICS treatment. Modelled probabilities of an exacerbation varied between 31 and 82% during the first year.

**Conclusions:**

The probability of an exacerbation was considerably higher than for pneumonia. ICS reduced exacerbations but did not influence the effect of risks associated with prior exacerbation history, GOLD stage, or female sex. The only identified risk factor for ICS-induced pneumonia was BMI <25 kg/m^2^. Analyses of this type may help the development of COPD risk equations.

## Background

Patients with chronic obstructive pulmonary disease (COPD) are at risk of exacerbations [1] and pneumonia [2, 3] and the reduction of future exacerbation risk has become an important treatment objective [4]. COPD management is therefore becoming like that of ischemic heart disease, where the aim is to reduce future risk as well as relieve current symptoms. The management of ischemic heart disease benefits from the use of predictive equations [5, 6], which use readily available clinical parameters to estimate risk of major cardiac events over the following 10 years. Similar risk equations may be helpful in COPD management.

Demonstrated risk factors for COPD exacerbations include low forced expiratory volume in 1 s (FEV_1_), current smoking, and a history of previous exacerbations [7–9], although another study found no association between FEV_1_ and COPD exacerbation risk [10]. Factors associated with increased pneumonia risk in COPD include: airway obstruction [11], low body mass index (BMI) [12, 13], older age [12, 14, 15], use of psychoanaleptics [11], presence of gastroesophageal reflux disease [16], increased blood neutrophil counts [17], and use of inhaled corticosteroids (ICS) [12, 18–21]. The effect of single factors on the risk of COPD exacerbations and pneumonia has been explored [12, 14, 15]. Although some individual factors may be relevant, the analysis of multiple factors seems more applicable [22]. However, it is not well understood how risk factors for exacerbations and pneumonia may interact, and whether, by evaluating multiple factors in combination, more precise probability estimates of future risk of exacerbation or pneumonia may be produced. If this were possible, it might allow the development of risk prediction equations for COPD patients treated with ICS-containing therapies.

This post-hoc analysis was designed to evaluate risk factors for moderate and/or severe exacerbations and pneumonia in COPD patients treated with ICS. An individual-patient pooled analysis approach was used, combining data from multiple studies to improve estimates of the size of the effect.

## Methods

### Data sources/studies included

Studies sponsored by GlaxoSmithKline plc with individual patient data were accessed. These were all randomized, parallel-group, double-blind, clinical trials of at least 52 weeks’ duration in COPD patients treated with the combination of the ICS fluticasone furoate with the long-acting β_2_ agonist (LABA) vilanterol, or fluticasone propionate plus salmeterol combination. They were required to have a LABA-alone treatment arm, a constant dose of ICS and a minimum of 100 patients per treatment arm, to ensure a sufficient number of events. Studies conducted prior to the TORCH study [23] were excluded, since there may have been a difference in the awareness of investigators of studies about the risk of pneumonia before and after that study, since it was the first to show that ICS-containing regimens are associated with an increased risk of pneumonia. Pneumonia events were identified using adverse event reports, standardised to the same version of the Medical Dictionary for Regulatory Activities (MedDRA) and list of adverse events of special interest (AESI). Finally, the studies had to have included patients with both moderate and severe/very severe airflow limitation, to be more representative of the population for whom ICS/LABA treatment is indicated.

All patients had provided written informed consent to participate in the included studies and all had been conducted in accordance with the Declaration of Helsinki, Good Clinical Practice guidelines, and ethical review requirements of participating institutions.

### Covariates examined

Only those covariates that were measured in all contributing studies were included in these analyses. We first examined a set of seven covariates that had previously been used in separate analyses of the individual studies: age (<65/≥65 years), BMI (<25/≥25 kg/m^2^), Global Initiative for Chronic Obstructive Lung Disease (GOLD) grade (I and II: pre-bronchodilator FEV_1_ ≥50%; III and IV: pre-bronchodilator FEV_1_ <50% [4]), number of exacerbations in the prior year (<2/≥2), history of smoking (former/current smoker), sex (male/female), study treatment (with/without ICS). This was termed the “small covariate set.”

We then repeated the analyses with an expanded set of nine covariates (“large covariate set”), including race (Asian/non-Asian) and World Bank Country Income Group [24]. The World Bank categorizes countries into four groups: high income (2017 gross national income per capita [GNIPC] ≥$12,056), upper-middle income (GNIPC $3896–$12,055), lower-middle income (GNIPC $996–$3895), and low income (GNIPC ≤$995) [24]; however, for this study these were regrouped further into two groups (high/non-high income) to give larger sample sizes in each category. The aim of including race and income groups was to try to account for possible differences in standards of care and reporting of pneumonia and exacerbations across different countries and ethnic backgrounds [25].

We refer to these individual covariates as main effects, but we also examined interaction effects, using all possible pairwise combinations of the main effects. There were 21 pairwise interactions derived from the small covariate set, and 36 pairwise interactions from the larger set. Neither study nor region were used as covariates; study was excluded since it would have no predictive value for the wider COPD population, and region was not used owing to collinearity problems with race.

### Backward selection

Time to first exacerbation and time to first pneumonia were analysed separately. A backward elimination process was used to identify the covariates that best explained the data: first a Cox proportional hazards model containing all main and interaction effects was fitted (21 pairwise interactions and 7 main effects in the smaller set; 36 pairwise interactions and 9 main effects in the larger set). The least statistically significant main or interaction effect was then removed from the model until all remaining main or interaction effects were significant (*p* < 0.1, which is standard for this type of analysis). Main effects, however, were not removed from the model if they were present in an interaction effect that remained in the model.

### Subgroup probabilities and hazard ratios

Once the final models were selected, the model-estimated probability of an event during the first year was shown for each subgroup combination of the covariates remaining in the model. The probabilities and hazard ratios (HRs) from the four final models – time to first pneumonia (smaller set), time to first pneumonia (larger set), time to first exacerbation (smaller set), and time to first exacerbation (larger set) – are presented.

## Results

Ten studies were identified and screened for eligibility. Five were excluded because they: lacked a LABA arm [26]; pre-dated TORCH [27, 28]; had <100 patients per arm [29]; included only patients with moderate COPD [30, 31]. Five studies were therefore identified for inclusion (Table 1). Demographic data for the contributing studies have been published [12, 32, 34] and are summarised in Table 2. Overall, 67% of patients included in the study were male, 54% had a BMI ≥25 kg/m^2^, 57% were former smokers and 54% were aged ≥65 years. The ICS-treated and non-ICS treated groups were generally well balanced (Table 2).

**Table 1:** Numbers of patents in the intent-to-treat (ITT) population and assigned treatment arms in the five contributing studies and the current meta-analysis

Study	FF/VI 200/25	FF/VI 100/25	FF/VI 50/25	VI 25	FP/SAL 250/50	SAL 50	FP 500	Placebo	Total: ITT ICS treated/non-ICS treated
Dransfield et al, 2013 [32] NCT01009463	402	403	408	409	–	–	–	–	1622 1213/409
Dransfield et al, 2013 [32] NCT01017952	409	403	412	409	–	–	–	–	1633 1224/409
Anzueto et al, 2009 [33, 34] NCT00115492	–	–	–	–	394	403	–	–	797 394/403
Ferguson et al, 2008 [35] NCT00144911	–	–	–	–	394	388	–	–	782 394/388
Crim et al, 2009 [12] NCT00268216	–	–	–	–	1533	1521	1534	1524	6112 3067/3045
Pooled analysis	811	806	820	818	2321	2312	1534	1524	10,946 6292/4654

*FF/VI* fluticasone furoate /vilanterol, *FP/SAL* fluticasone/salmeterol, *ICS* inhaled corticosteroids

**Table 2:** Summary of demographic characteristics in the current meta-analysis

Study	ICS-treated patients (*N* = 6292)	Non-ICS treated patients (*N* = 4654)	Total (*N* = 10,946)
Sex, *n* (%)			
Female	2157 (34)	1425 (31)	3582 (33)
Male	4135 (66)	3229 (69)	7364 (67)
Mean age, years (SD)^a^	64.5 (8.80)	64.8 (8.56)	64.7 (8.70)
Age group, *n* (%)^a^			
≤ 64years	2945 (47)	2114 (45)	5059 (46)
≥65 years	3347 (53)	2540 (55)	5887 (54)
BMI subgroup, *n* (%)			
<25 kg/m^2^	2848 (45)^†^	2217 (48)^‡^	5065 (46)^§^
≥25 kg/m^2^	3442 (55)^†^	2435 (52)^‡^	5877 (54)^§^
History of smoking, *n* (%)			
Current smoker	2720 (43)**	1991 (43)	4711 (43)^††^
Former smoker	3571 (57)**	2663 (57)	6234 (57)^††^

*BMI* body mass index, *ICS* inhaled corticosteroids, *SD* standard deviation

^a^Age was imputed when full date of birth was not provided, ^†^*N* = 6290, ^‡^*N* = 4652, ^§^*N* = 10,942, **N = 6291, ^††^*N* = 10,945

Inclusion/exclusion criteria and the definitions used for moderate and severe exacerbations for each study are summarised in Additional file 1 Pneumonia model.

**Fig. 1:**
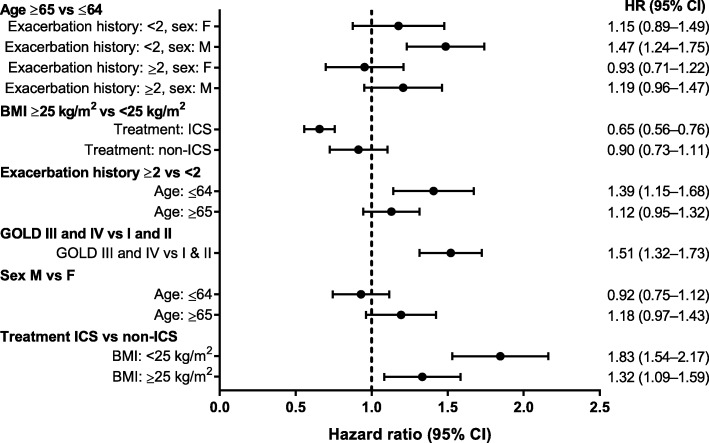
Hazard ratios (95% confidence intervals [CIs]) for pneumonia from selected seven-covariate pneumonia model. *BMI* body mass index, *GOLD* Global Initiative for Chronic Obstructive Lung Disease, *ICS* inhaled corticosteroids

In the “small covariate set,” a low BMI (<25 kg/m^2^), history of ≥2 exacerbations, worse lung function (GOLD grades III and IV) and treatment with ICS appeared to be important factors increasing the risk of pneumonia (Fig. 1). In summary: pneumonia risk was higher in men aged ≥65 with a history of <2 exacerbations than younger men. In addition, the risk of pneumonia associated with an exacerbation history (≥2 vs <2) was higher for younger patients (≤64 years) than for older patients (≥65 years). Patients with lower BMI (<25 kg/m^2^) were at greater risk of pneumonia on ICS treatment than those with higher BMI, but ICS treatment did not alter the risk associated with any of the other factors.

**Fig. 2:**
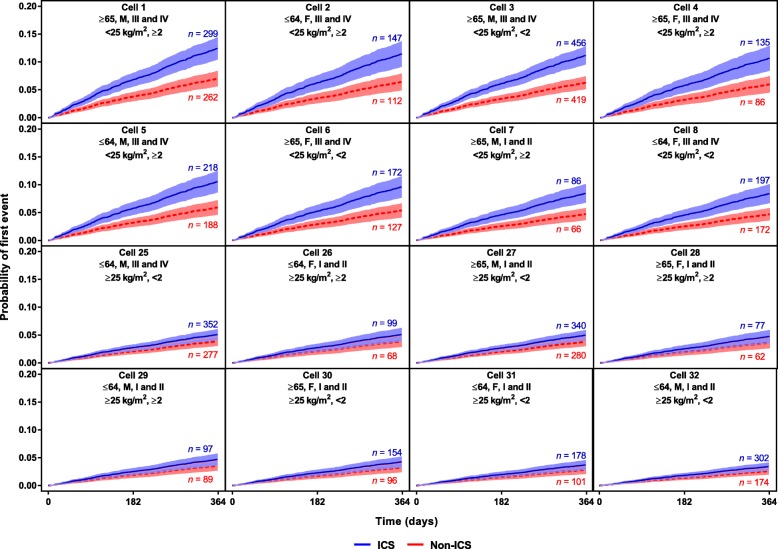
Survival curves (95% CI bands) from selected pneumonia model subgroups showing probability of first pneumonia during year on study treatment. Cell header line 1: Age (years), sex, Global Initiative for Chronic Obstructive Lung Disease stage; Cell header line 2: Body mass index, number of exacerbations in the prior year. Numbers of patients presented are subgroup numbers; patients without covariates did not contribute to the model. All cells are shown in S1. *CI* confidence interval, *ICS* inhaled corticosteroids

The modelled probability of pneumonia varied between 3 and 12% during the first year of the studies (the only common time period) in the identified subgroups (Fig. 2; Additional file 2). The most at-risk subgroups were generally older, with a low BMI, a history of exacerbations, worse lung function, and treated with ICS.

**Fig. 3:**
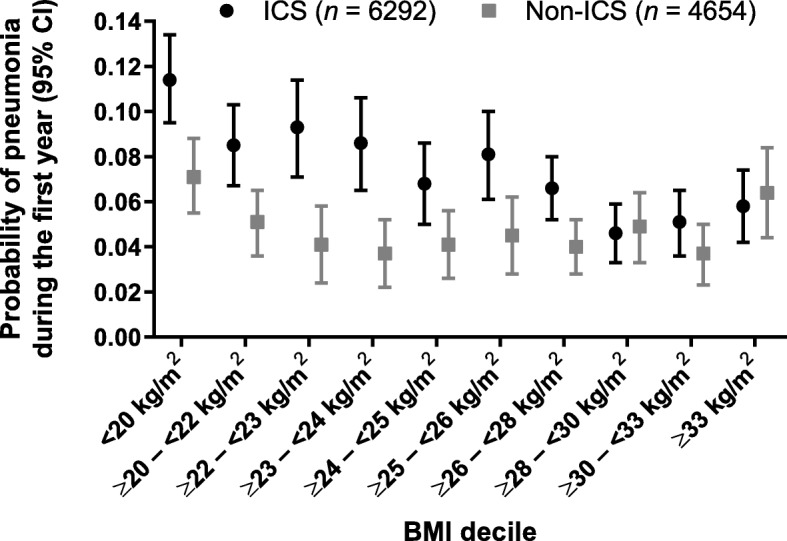
Probabilities (95% CIs) of pneumonia during first year by BMI decile (direct adjusted probabilities). *BMI* body mass index, *CI* confidence interval, *ICS* inhaled corticosteroids

To further investigate how BMI modifies the pneumonia risk associated with treatment with ICS, we ran the model for a second time using a BMI covariate that had 10 levels (rough deciles), and present the direct adjusted probabilities of pneumonia during the first year in each BMI by treatment group (ICS vs non-ICS) (Fig. 3). This shows evidence of a U-shaped curve, with an increased risk of pneumonia at low and high BMIs in the absence of ICS treatment. A separation between treatment groups was particularly apparent for patients with BMI <24 kg/m^2^.

The final model using the “large covariate set” provided no additional insight, since most of the patients were of non-Asian ethnicity and from the high-income subgroup (Additional file 3). It qualitatively replicated the findings from the “small covariate set,” and all terms in the model from the small set were retained in the model from the large set. Income group, race, and various interactions including these terms also remained in the “large covariate set”. Income and race did not affect pneumonia in a qualitatively consistent manner.

### Exacerbation model

**Fig. 4:**
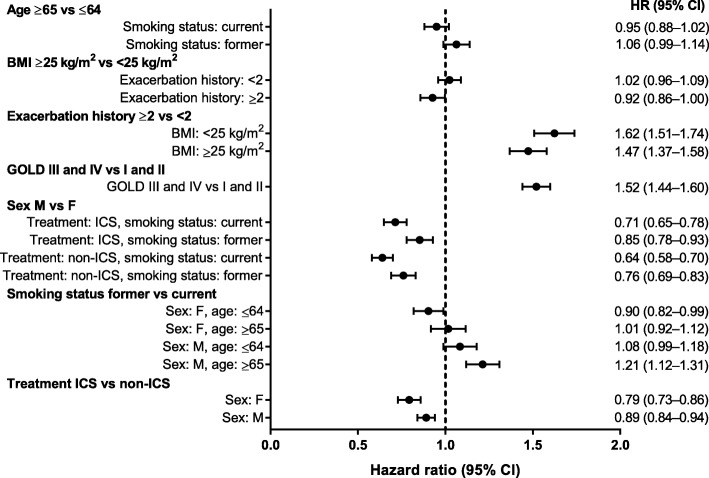
Hazard ratios (95% CIs) for exacerbation from selected seven-covariate exacerbation model. *BMI* body mass index, *GOLD* Global Initiative for Chronic Obstructive Lung Disease, *ICS* inhaled corticosteroids

**Fig. 5:**
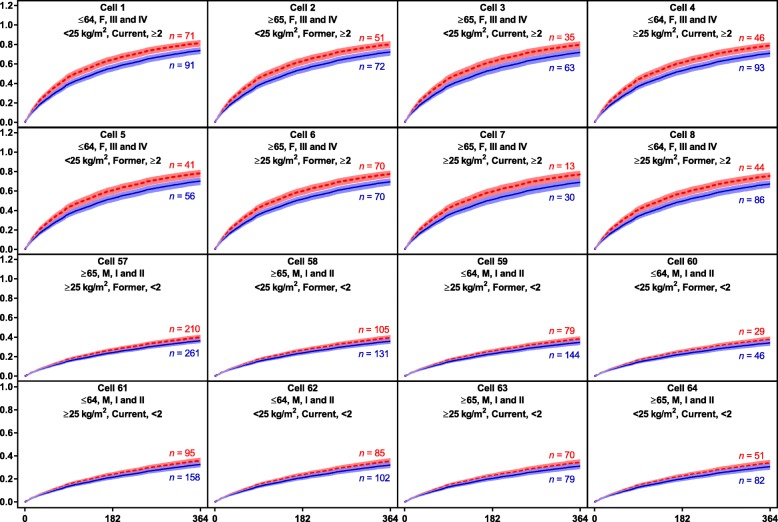
Survival curves (95% CI bands) from selected exacerbation model subgroups showing probability of first exacerbation during year on study treatment. Cell header line 1: age (years), sex, Global Initiative for Chronic Obstructive Lung Disease stage; Cell header line 2: Body mass index, number of exacerbations in the prior year. Numbers of patients presented are subgroup numbers; patients without covariates did not contribute to the model. *CI* confidence interval, *ICS* inhaled corticosteroids

In the “small covariate set,” lack of a history of exacerbations (<2 exacerbations), better lung function (GOLD grades I and II), male sex, and ICS treatment appeared to be important factors associated with a lower exacerbation risk (Fig. 4). The modelled probability of an exacerbation varied between 31 and 82% during the first year in the identified subgroups. The most at-risk subgroups were generally female, with lower BMI (<25 kg/m^2^), a history of exacerbations (≥2), and worse lung function (GOLD stages III and IV) (Fig. 5).

The final model from the “large covariate set” differed somewhat from the model selected from the “small covariate set”: of the interactions found above, only two were replicated. These were sex by smoking status (reduction in exacerbation risk for males vs females was smaller for former smokers) and BMI by exacerbation history (the increase in exacerbation risk for patients with a history of exacerbations was greater in patients with a lower than higher BMI).

### Exacerbations in the pneumonia model

**Fig. 6:**
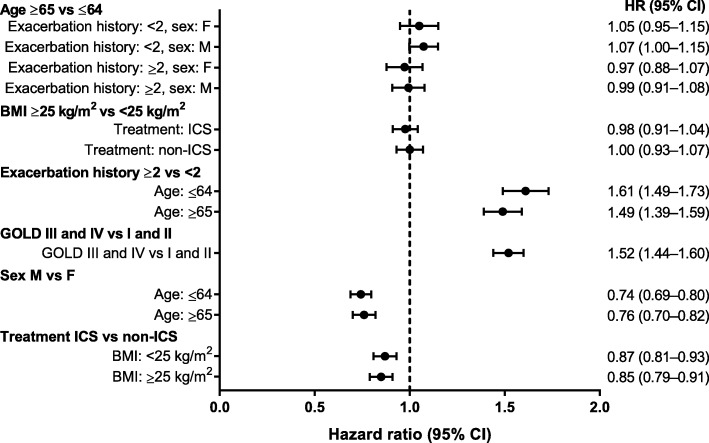
Hazard ratios (95% CIs) for exacerbation from selected Cox pneumonia model. *BMI* body mass index, *GOLD* Global Initiative for Chronic Obstructive Lung Disease, *ICS* inhaled corticosteroids

**Fig. 7:**
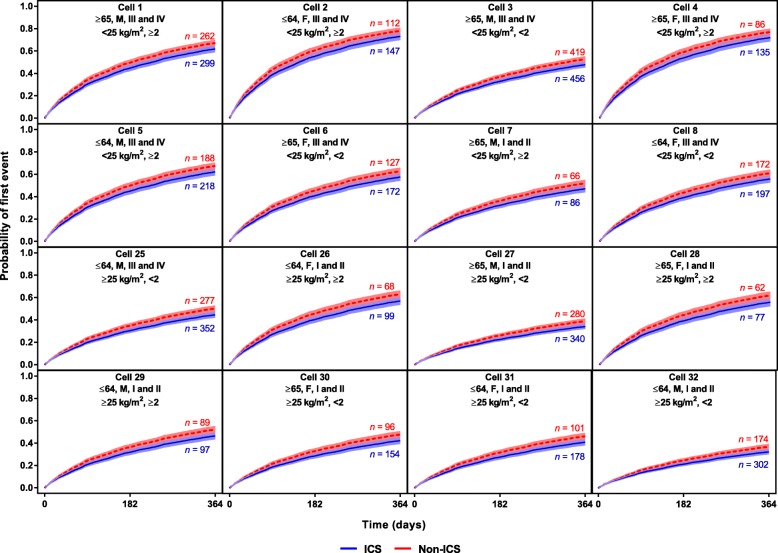
Survival curves (95% CI bands) from selected pneumonia model subgroups showing probability of first exacerbation during year on study treatment. Cell header line 1: age (years), sex, Global Initiative for Chronic Obstructive Lung Disease stage; Cell header line 2: Body mass index, number of exacerbations in the prior year. Numbers of patients presented are subgroup numbers; patients without covariates did not contribute to the model. All cells are shown in S2. *CI* confidence interval, *ICS* inhaled corticosteroids

The background to this analysis is a desire to provide better estimates of possible risk (pneumonia) and possible benefit (exacerbation reduction), so we tested how the subgroups selected in the pneumonia model behaved in terms of prediction of exacerbations. Age and BMI appeared to have little effect on exacerbation risk. HRs associated with exacerbation history (≥2 vs <2) appeared to be larger for exacerbation risk than pneumonia risk. The HR associated with lung function (GOLD grades III/IV vs I/II) appeared to be similar for exacerbation risk and pneumonia risk. Patients with a BMI <25 kg/m^2^ treated with ICS versus non-ICS had an HR of 0.87 (95% confidence interval [CI], 0.81–0.93) for exacerbations (Fig. 6) and 1.83 (95% CI, 1.54–2.17) for pneumonia (Fig. 1). Patients with a BMI ≥25 kg/m^2^ treated with an ICS versus non-ICS had an HR of 0.85 (95% CI, 0.79–0.91) for exacerbations (Fig. 6) and 1.32 (95% CI, 1.09–1.59) for pneumonia (Fig. 1). Corresponding probabilities are presented in Fig. 7 and Additional file 4.

## Discussion

This analysis explored the interaction between risk factors for exacerbations and pneumonia in COPD patients. The results show that modelled probabilities of an exacerbation over a 1-year period were considerably higher than those for pneumonia (31–82% vs 3–12%, respectively).

The only risk factor for pneumonia influenced by ICS treatment was BMI, with ICS treatment further increasing risk for patients with BMI <25 kg/m^2^. A BMI of <25 kg/m^2^ has been reported as a pneumonia risk factor for a study population with COPD and moderate airflow limitation, along with a prior history of exacerbations and FEV_1_ <60% of predicted [36], and our findings extend this observation across a broader range of airflow limitation. In the general population, an increased risk of pneumonia or respiratory infection has been associated both with obesity [37] and, in women, with being underweight [38]. Obesity has also been reported as a risk factor for influenza/influenza-like disease in patients undergoing seasonal influenza vaccination [39]. In COPD, reduced exacerbation frequency has been described in patients with a BMI ≥24 kg/m^2^ [40] and ≥25 kg/m^2^ [41], although there does not seem to be a clear association between observed BMI and exacerbations [42]. There have been reports of both low and high BMI being associated with an increased risk of pneumonia. An association has been described between increased risk of community-acquired pneumonia (CAP) in patients with lower BMI (median BMI for no CAP was 23.7 and for CAP was 22.7) [43], while another study found significantly increased risk of pneumonia in patients with COPD and a BMI ≥35 kg/m^2^ [42]. Our study reported a possible U-shaped effect in patients with COPD, with pneumonia risk appearing to be increased for patients with the lowest and highest BMIs. This trend was also observed in the Copenhagen General Population Study, with an increased risk of pneumonia for patients with a BMI <18.5 kg/m^2^, although this was not significant due to low numbers [42]. Similarly, a U-shaped relationship has previously been reported for BMI and risk of influenza-related pneumonia from a large meta-analysis in children and adults [44].

When exacerbations were modelled using the same subgroups found in the model that best explained pneumonia risk, BMI did not appear to significantly influence the ICS treatment effect on exacerbations. Thus, it appears that patients with BMI <25 kg/m^2^may have a lower ratio of benefit to risk from ICS treatment than those with BMI ≥25 kg/m^2^. It is possible that the identified ICS by BMI interaction was driven by the Asian population in our analysis, since it had a lower BMI and there are reported differences in ICS inhaled clearance in Asian populations compared with those of White/Caucasian ethnicity [45]. However, our models could not include a 3-way interaction (BMI*ICS*Asian ethnicity) due to an insufficient number of events, so these relationships will need to be tested in larger datasets.

Of the interactions found to influence the pneumonia risk in our analysis: age by sex (*p* = 0.078), age by exacerbation history (*p* = 0.096), and treatment with ICS by BMI (*p* = 0.012), the most significant and clinically plausible appears to be the interaction between BMI and treatment with ICS. When the same covariates were tested as predictors of exacerbations, none of these interactions were significant at the 5% level. Using the exacerbation model from the “small covariate set”, the most significant interaction was sex by smoking status (*p* < 0.001), which was also significant in the “large covariate set.” Similarly, the exacerbation history by BMI interaction (*p* = 0.052) in the “small covariate set” was also identified in the “large covariate set,” but other significant interactions were not replicated.

The strength of this analysis is the novel exploration of interactions between risk factors for pneumonia and exacerbations in a large sample size. Full individual-patient data could be accessed, so there was no bias at the individual study level in terms of outcomes.

There are, however, limitations associated with the baseline covariates collected in the individual studies. Only those covariates measured in all contributing studies could be included, so this did not, for example, allow inclusion of biomarkers like eosinophils. The analysis was also retrospective in nature and the contributing studies were conducted in different regions and at different times. Furthermore, the nature of repeated testing in the backward elimination procedure does not conserve the type I error, so results should be considered hypothesis generating. By using binary covariates, we may have lost information about the subtleties of the effects; however, binary covariates are simpler to interpret than multilevel covariates, and unlike the use of continuous variables they require no assumptions about the linearity of effect size. Interpretation of correlated covariates must be done with care, and binary covariates do not fully adjust for all effects related to that covariate. When pooling data from different studies, “study” as a covariate is generally used to account for variability in effect sizes from unmeasured covariates that may affect the different studies; however, due to the study covariate likely having little predictive value in a wider population of COPD patients, not recruited to a clinical trial, we omitted this variable. As a result, we could not account for any differences that it may measure. We assume that the race and country income covariates used in the sensitivity analyses with the “large covariate set” go some way toward accounting for the type of differences that a study covariate would measure.

Our results should not be considered as providing a validated risk equation because we did not split the dataset into two groups: one to develop the model and one to test it. This was largely due to the relatively small number of pneumonia events, despite the large number of participants in the pooled dataset. Further work should use individual patient-level data from a broader range of studies, with a wider range of covariates including biomarkers, and not be restricted to trials sponsored by a single pharmaceutical company.

## Conclusions

The hypothesis-generating methods used for this analysis may be applicable to future analyses designed to develop risk equations for exacerbation benefit and treatment-associated pneumonia risk in COPD. They will require testing and validation in other, larger datasets.

## Supplementary information

**Additional file 1.** Summary of key inclusion and exclusion criteria, the definition of a COPD exacerbation, and pneumonia adverse event assessment information from the studies in this analysis.

**Additional file 2.** Survival curves (95% CI bands) from selected pneumonia model showing probability of first pneumonia during year on study treatment.

**Additional file 3.** Summary of patients in the race and country income subgroups (based on the nine-covariate model).

**Additional file 4.** Survival curves (95% CI bands) from selected pneumonia model showing probability of first exacerbation during year on study treatment.

## Data Availability

All clinical study reports from the individual studies included in this analysis, the protocol/analysis plan for this meta-analysis and the results of this analysis are publicly available at https://www.gsk-clinicalstudyregister.com. Additional data may be requested for research proposals. Proposals approved by an independent review committee should be submitted to www.clinicalstudydatarequest.com. A data access agreement will be required.

## Abbreviations

BMI: Body mass index
COPD: Chronic obstructive pulmonary disease
FEV1: Forced expiratory volume in 1 s
GNIPC: Gross national income per capita
GOLD: Global Initiative for Chronic Obstructive Lung Disease
HR: Hazard ratio
ICS: Inhaled corticosteroid
LABA: Long-acting β2 agonist
TORCH: TOwards a Revolution in COPD Health [study]
VI: Vilanterol
