# Supplementary material for: Risk factors for exacerbations and pneumonia in patients with chronic obstructive pulmonary disease: a pooled analysis
Source: Respir Res. 2020 Jan 6;21:5. doi: 10.1186/s12931-019-1262-0 (PMC6945447; doi:10.1186/s12931-019-1262-0)
Supplement: Supplementary file 1 — Additional file 1. Summary of key inclusion and exclusion criteria, the definition of a COPD exacerbation, and pneumonia adverse event assessment information from the studies in this analysis. [file 12931_2019_1262_MOESM1_ESM.docx]

**Additional file 1.** Summary of key inclusion and exclusion criteria, the definition of a COPD exacerbation, and pneumonia adverse event assessment information from the studies in this analysis.

| Study | NCT01009463^1,2^ | NCT01017952^1,3^ | | NCT00144911^4,5,6^ | | NCT00115492^4,7,8^ | | | NCT00268216^9,10,11,12^ |
| --- | --- | --- | --- | --- | --- | --- | --- | --- | --- |
| Key inclusion criteria, at screening  Outpatients  Male or female  Aged ≥40 years  Diagnosed COPD  Current or prior smoking history of ≥10 pack years  FEV_1_/FVC ratio ≤0.70  FEV_1_ % of predicted normal value  ≥1 moderate or severe COPD exacerbation in the   prior 12 months | Yes  Yes  Yes  Yes*  Yes**^†^**  Yes, post BD  Yes, post BD >70%  Yes ^‡^ | Yes  Yes  Yes  Yes*  Yes**^†^**  Yes, post BD  Yes, post BD <70%  Yes ^‡^ | | -  Yes  Yes  Yes  Yes  Yes, pre BD  Yes, pre BD ≤50%  Yes | | -  Yes  Yes  Yes  Yes  Yes, pre BD  Yes, pre BD ≤50%  Yes | | | Yes  Yes  Yes, 40–80  Yes  Yes  Yes, pre BD  Yes, pre BD ≤60%  - |
| Key exclusion criteria, at screening  Diagnosis of asthma or other respiratory disorders^§^  Requirement for long-term oxygen therapy  Concurrent use of medications  Lung resection surgery in the prior 12 months^¶¶^  Moderate/severe COPD exacerbation  History of/current significant health conditions that   could affect evaluation of safety/effectiveness  Protocol violations, missing data or non-compliance  Evidence of alcohol, drug or solvent abuse  Hypersensitivity to any study medications  Prior use of investigational drug/study medication | Yes**^¶^**  Yes**^††^**  Yes^‡‡^  Yes  Yes, if ≥14 days prior to screening ≥30 days post OCS  Yes**^†††^**  Yes**^¶¶¶^**  Yes  Yes  Yes | Yes**^¶^**  Yes**^††^**  Yes^‡‡^  Yes  Yes, if ≥14 days prior to screening ≥30 days post OCS  Yes**^†††^**  Yes**^¶¶¶^**  Yes  Yes  Yes | | Yes**  -  Yes^§§^  Yes  Yes, if not resolved prior to screening  Yes^‡‡‡^  Yes  -  -  - | | Yes**  -  Yes^§§^  Yes  Yes, if not resolved prior to screening  Yes^‡‡‡^  Yes  -  -  - | | | Yes  Yes  Yes, OCS  Yes***  Yes, if during run in period  Yes^§§§^  ^-^  Yes  Yes  In prior 4 weeks |
| COPD exacerbation definitions used in each study  Moderate  Severe | Necessitating treatment with OCS and/or antibiotics.  Necessitating hospital admission. | Necessitating treatment with OCS and/or antibiotics.  Necessitating hospital admission. | | Requiring outpatient antibiotic and/or OCS use.  Requiring hospitalisation. | | Requiring outpatient antibiotic and/or OCS use.  Requiring hospitalisation. | | | Requiring treatment with systemic corticosteroids and/or antibiotics.  Requiring hospitalisation. |
| Pneumonia AE assessments in each study | Pneumonia assessed as distinct safety endpoint.  Chest radiographs to be taken within 48 h of suspected pneumonia or a moderate/severe exacerbation.  Radiographs over-read by a central laboratory; event recording as pneumonia or an exacerbation was at investigators' discretion.  Pneumonia events reported as AEs and those radiographically confirmed are reported.^1^ | Pneumonia assessed as distinct safety endpoint.  Chest radiographs to be taken within 48 h of suspected pneumonia or a moderate/severe exacerbation.  Radiographs over-read by a central laboratory; event recording as pneumonia or an exacerbation was at investigators' discretion.  Pneumonia events reported as AEs and those radiographically confirmed are reported.^1^ | | Safety was assessed by adverse event reporting.^6^ | | Safety was assessed by adverse event reporting.^8^ | | | There were no protocol-defined criteria for pneumonia, nor were chest radiographs, sputum cultures or laboratory evaluations required to confirm the clinical diagnosis.  In addition, no specific queries were made to the investigators with respect to pneumonia. Investigators recorded all AEs, including pneumonia reports, in the case report form. ^12^ |
|  |  | |  | |  | |  |  | |

*Patients with a clinical history of COPD in accordance with the following definition by the American Thoracic Society/European Respiratory Society.^13^
**^†^**Number of pack years = (number of cigarettes per day/20) x number of years smoked.
^‡^A documented history (e.g., medical record verification) was required; prior use of antibiotics alone did not qualify as an exacerbation history unless the use was associated with treatment of worsening symptoms of COPD, such as increased dyspnea, sputum volume, or sputum purulence (color).
^§^Including α1-antitrypsin deficiency as the underlying cause of COPD.
**^¶^**Including active tuberculosis, lung cancer, bronchiectasis, sarcoidosis, lung fibrosis, pulmonary hypertension, interstitial lung diseases or other active pulmonary diseases; pneumonia or risk factors for pneumonia immune suppression (HIV, Lupus, etc) or other risk for pneumonia (e.g. neurological disorders affecting control of the upper airway, such as Parkinson's, Myasthenia Gravis, etc).
**Asthma diagnosis according to American Thoracic Society standards.
**^††^**Or nocturnal oxygen therapy required for ≥12 hours a day.
^‡‡^Patients medically unable to withhold their albuterol/salbutamol and/or ipratropium for the 4-hour period required prior to spirometry testing at each study visit and/or unable to stop using medications such as BDs and corticosteroids for protocol-specified times prior to screening were excluded. Patients in the acute phase of a PRP within 4 weeks prior to screening or who would enter the acute phase of a PRP during the study were excluded; patients in the maintenance phase of a PRP were not excluded.
^§§^Long-acting beta-agonists, long-acting anticholinergics, ICS and OCS, theophylline, investigational medications, ritonavir, and anti-leukotrienes.
**^¶¶^**Or volume reduction surgery.
***And/or lung transplantation.
**^†††^**Including clinically significant: abnormalities not believed to be due to the presence of COPD revealed by a Chest X-ray or CT scan, uncontrolled hypertension or peptic ulcer disease, carcinoma not complete remission for ≥5 years, sleep apnea requiring use of continuous positive airway pressure device or non-invasive positive pressure ventilation device, cardiovascular (i.e., pacemaker), neurological, psychiatric, renal, hepatic, immunological, endocrine (including uncontrolled diabetes or thyroid disease) or uncontrolled haematological abnormalities. Pregnant patients and those with questionable validity of consent were also excluded.
^‡‡‡^A history or current significant health conditions that could affect patient safety or effectiveness evaluation if the condition exacerbates during the study, such as cardiac arrhythmias, uncontrolled/unstable congestive heart failure, uncontrolled hypertension or unstable angina, including abnormal and clinically significant ECG findings.
^§§§^Other conditions likely to interfere with the study or cause death within 3 years.
**^¶¶¶^**Including affiliation with the investigator site.

*AE* adverse event, *BD* bronchodilator (albuterol/salbutamol), *COPD* chronic obstructive pulmonary disease, *CT* computed tomography, *ECG* echocardiogram, *FEV_1_* forced expiratory volume in 1 second, *FVC* forced vital capacity, *HIV* human immunodeficiency virus, *ICS* inhaled corticosteroid, *OCS* oral corticosteroid, *PRP* pulmonary rehabilitation program. - not mentioned on NCT study listing or in cited manuscript.

**References:**

1. Dransfield MT, Bourbeau J, Jones PW, Hanania NA, Mahler DA, Vestbo J, et al. Once-daily inhaled fluticasone furoate and vilanterol versus vilanterol only for prevention of exacerbations of COPD: two replicate double-blind, parallel-group, randomised controlled trials. Lancet Respir Med. 2013;1:210–23.
2. Clinicaltrials.gov listing for study NCT01009463. <https://clinicaltrials.gov/ct2/show/NCT01009463>. Accessed 24 October 2019.
3. Clinicaltrials.gov listing for study NCT01017952. https://clinicaltrials.gov/ct2/show/NCT01017952. Accessed 24 October 2019.
4. Disantostefano RL, Li H, Rubin DB, Stempel DA. Which patients with chronic obstructive pulmonary disease benefit from the addition of an inhaled corticosteroid to their bronchodilator? A cluster analysis. BMJ Open. 2013;3. pii: e001838. doi: 10.1136/bmjopen-2012-001838.
5. Clinicaltrials.gov listing for study NCT00144911. https://clinicaltrials.gov/ct2/show/NCT00144911. Accessed 24 October 2019.
6. Ferguson GT, Anzueto A, Fei R, Emmett A, Knobil K, Kalberg C. Effect of fluticasone propionate/salmeterol (250/50 mcg) or salmeterol (50 mcg) on COPD exacerbations. Respir Med. 2008;102:1099–108.
7. Clinicaltrials.gov listing for study NCT00115492. https://clinicaltrials.gov/ct2/show/NCT00115492. Accessed 24 October 2019.
8. Anzueto A, Ferguson GT, Feldman G, Chinsky K, Seibert A, Emmett A, et al. Effect of fluticasone propionate/salmeterol (250/50) on COPD exacerbations and impact on patient outcomes. J COPD. 2009;6:320–9.
9. Calverley PM, Anderson JA, Celli B, Ferguson GT, Jenkins C, Jones PW, et al. Salmeterol and fluticasone propionate and survival in chronic obstructive pulmonary disease. N Engl J Med. 2007;356:775–89.
10. Vestbo J, TORCH Study Group. The TORCH (TOwards a Revolution in COPD Health) survival study protocol. Eur Respir J. 2004;24:206–210.
11. Clinicaltrials.gov listing for study NCT00268216. https://clinicaltrials.gov/ct2/show/NCT00268216. Accessed 24 October 2019.
12. Crim C, Calverley PM, Anderson JA, Celli B, Ferguson GT, Jenkins C, et al. Pneumonia risk in COPD patients receiving inhaled corticosteroids alone or in combination: TORCH study results. Eur Respir J. 2009;34:641–47.
13. Celli BR, MacNee W, Agusti A, Anzueto A, Berg B, Buist AS, et al. Standards for the diagnosis and treatment of patients with COPD: a summary of the ATS/ERS position paper. Eur Respir J. 2004;23:932–946.
