# Supplementary material for: Risk factors for exacerbations and pneumonia in patients with chronic obstructive pulmonary disease: a pooled analysis
Source: Respir Res. 2020 Jan 6;21:5. doi: 10.1186/s12931-019-1262-0 (PMC6945447; doi:10.1186/s12931-019-1262-0)
Supplement: Supplementary file 2 — Additional file 2. Survival curves (95% CI bands) from selected pneumonia model showing probability of first pneumonia during year on study treatment. [file 12931_2019_1262_MOESM2_ESM.pdf]

Additional file 2 Survival curves (95% CI bands) from selected pneumonia model showing probability of first pneumonia during year on study treatment

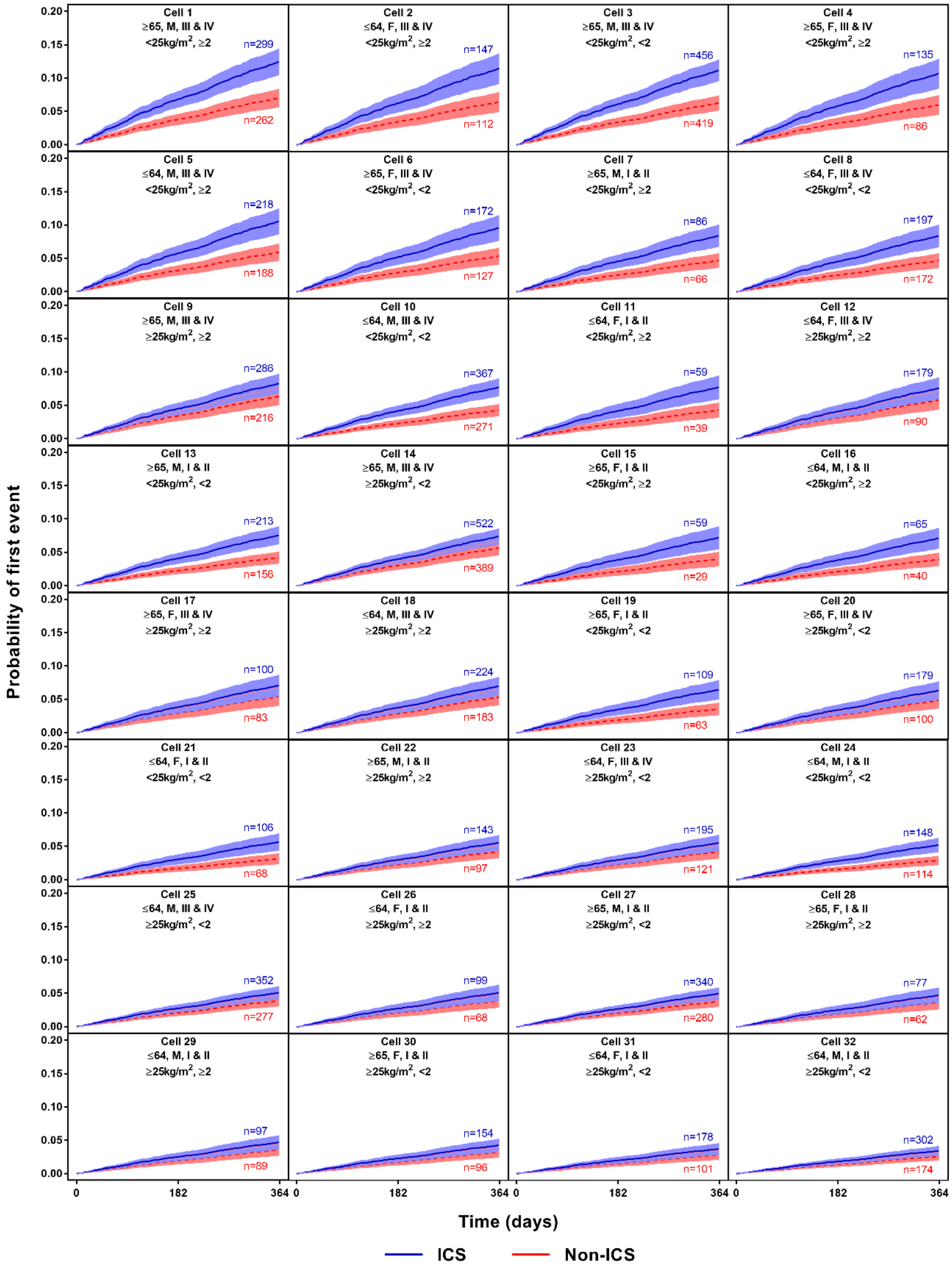

Cell header line 1: Age (years), gender, Global Initiative for Chronic Obstructive Lung Disease stage; Cell header line 2: Body mass index, number of exacerbations (<2, ≥2) in the prior year. Numbers of patients presented are subgroup numbers, patients without covariates did not contribute to the model. *CI* confidence interval, ICS inhaled corticosteroid
