# Supplementary material for: Risk factors for exacerbations and pneumonia in patients with chronic obstructive pulmonary disease: a pooled analysis
Source: Respir Res. 2020 Jan 6;21:5. doi: 10.1186/s12931-019-1262-0 (PMC6945447; doi:10.1186/s12931-019-1262-0)
Supplement: Supplementary file 3 — Additional file 3. Summary of patients in the race and country income subgroups (based on the nine-covariate model). [file 12931_2019_1262_MOESM3_ESM.docx]

**Additional file 3** Summary of patients in the race and country income subgroups (based on the nine covariate model)

| Subgroup, n (%) | ICS  (*N* = 6292) | Non-ICS  (*N* = 4654) | Total  (*N* = 10,946) |
| --- | --- | --- | --- |
| Race  Asian  Non-Asian  Missing | 518 (8)  5773 (92)  1 (<1) | 428 (9)  4225 (91)  1 (<1) | 946 (9)  9998 (91)  2 (<1) |
| Country income  Non-high income*  High income | 1811 (29)  4481 (71) | 1310 (28)  3344 (72) | 3121 (29)  7825 (71) |

*Consists of those classed as upper-middle, lower-middle, and low income groups. *ICS* inhaled corticosteroid
